# Comparison of pain between bilateral ICL surgeries in patients with myopia

**DOI:** 10.1186/s12886-024-03450-5

**Published:** 2024-04-16

**Authors:** Yu Xiao, Yali Liu

**Affiliations:** West China School of Public Health and West China Fourth Hospital,Sichuan University, Chengdu, Sichuan China

**Keywords:** Intraoperative pain, Implantable collamer lens surgery , MCP-1, Intraoperative cooperation, Surgical satisfaction, Bilateral surgical interval

## Abstract

**Purpose:**

The purpose of this study was to compare the preoperative anxiety, aqueous humor monocyte chemoattractant protein-1 (MCP-1) concentration, intraoperative pain, and degree of cooperation of the first eye implantable collamer lens (ICL) surgery with the second eye surgery, of the 1-day interval group with the 1-week interval group, and to investigate the possible causes of these differences, as well as to determine the appropriate interval between bilateral eye ICL surgeries.

**Method:**

The study was a prospective observational study. A total of 120 patients who underwent bilateral ICL surgery at the Department of Ophthalmology, West China Fourth Hospital, Sichuan University, from July to September 2023, were enrolled. The patients were divided into a 1-day interval group and a 1-week interval group. The ICL surgery was performed on both eyes according to the schedule. Anxiety levels, aqueous humor MCP1, cooperativeness, surgical time, pain and satisfaction, and patients’ estimations of the time spent in the operation were recorded for each eye. The patients were instructed to recall the intraoperative pain of the first eye surgery after the operation of the second eye. Statistical analyses (two independent samples t-test,two paired samples t-test, the rank-sum test, the chi-square test, non-parametric test with multiple independent samples) were performed to compare the differences between each score in both eyes and two groups. Furthermore, we examined the relationship between pain levels and the reproductive history of the patients.

**Results:**

In the 1-day interval group, male/female is 22/52, average age is 25.24±5.00. In the 1-week interval group, male/female is 17/29, average age is 25.39±5.57. There was no statistically significant difference between the two groups. In both groups, patients were less nervous, had significantly more pain, had less surgical satisfaction, had a longer estimated operative time, and had elevated preoperative MCP1 during the second eye operation. In the second eye surgery, the patient’s cooperation worsened, but it did not lead to an increase in surgical time. A significant proportion of patients, particularly in the 1-week interval group, recalled experiencing reduced pain during the first eye surgery. The 1-week interval group had a higher difference in all indicators between the bilateral surgeries. In the second eye surgery, patients in the 1-week interval group experienced more severe pain, less cooperation, longer estimated operation duration, and a greater MCP1 than those in the 1-day interval group.

**Conclusion:**

Patients undergoing second eye ICL surgery had decreased nervousness, increased pain, decreased cooperation, and satisfaction, and increased MCP1 compared to the first eye surgery. It is recommended that an interval of about one week should be avoided between bilateral surgeries when developing a surgical schedule to improve patients’ cooperation, satisfaction, and comfort.

## Introduction

With the widespread use of devices such as mobile phones and tablets, the prevalence of myopia is increasing. Researchers estimate that in 30 years, the global myopia prevalence will reach nearly 50%, while high myopia prevalence will reach nearly 10% [1]. Currently, keratorefractive laser surgery is the mainstream method for treating myopia, but some patients are not suitable for the surgery due to their corneal conditions. There are also disadvantages, such as poor visual quality, poor predictability and irreversibility [2]. Implantable collamer lens (ICL) surgery is an effective method for treating myopia [3, 4]. It is a posterior chamber phakic intraocular lens that is a safe and effective surgical option for the correction of moderate to high myopia. It overcomes some of keratorefractive laser surgery’s limitations (unsuitable for too thin corneas and too high myopia) and is less constrained by corneal conditions and refractive power [5]. It also has advantages such as reversibility, less dry eye, fewer corneal complications, and better visual quality. Patients who undergo bilateral ICL surgery often complain about more pain after the second surgery in our clinical practice. Even though both eye surgeries are performed by the same physician, with the same anesthesia method, surgical instruments, and artificial lens implanted. Previous studies on cataract surgery have suggested that patients with bilateral cataracts often complain about more pain in the second eye surgery [6–9]. However, quantitative data on whether the second eye ICL surgery is more painful is currently lacking. MCP1, a new research area, has been shown to be an important cause of surgical pain. Previous studies on pain of cataract surgery have noted a positive correlation between MCP1 concentration in aqueous humor and pain of the surgery. In our study, we recorded the preoperative anxiety level, intraoperative pain, cooperation level, and surgical time, MCP-1 in patients who underwent bilateral ICL surgery to determine if the second eye surgery was more painful than the first and to investigate the underlying cause. Previous studies on cataracts have noted that the interval between binocular surgeries affects the pain of the second eye surgery. The association between the interval between bilateral procedures and the pain level during the second eye surgery was also explored to guide the interval selection.

## Data and methods

### General information and grouping

The study was a prospective observational study. 120 patients (39 males, 81 females) who underwent bilateral ICL surgery at West China Fourth Hospital of Sichuan University from July to September 2023 were enrolled. The Inclusion criteria: Adult myopic patients with indications [10, 11] for surgery in both eyes(the patient has the desire to improve the refractive status, and has a reasonable expectation of surgical efficacy;18-45 years old; patients with myopia or combined astigmatism; relative stability of refractive error, i.e. change in refractive error ≤0.50D per year for 2 consecutive years; corneal endothelium count ≥2000/mm^2 ^with stable cell morphology; depth of the anterior chamber ≥2.80mm and the angle of chamber is open), Mentally and psychologically normal ,Voluntary enrollment in this study. The exclusion criteria: Low ocular pain threshold(hyperalgesia, a heightened sensation of pain in response to a normal analgesic stimulus. Physical examination reveals an increased response to stimuli, e.g., finger pressure ,hot and cold stimuli, pinpricks can lead to significant and severe pain.), poor communication and comprehension, and cognitive abnormalities, Other ocular problems such as iritis, retinal fissure, and previous surgical history in the inner eye ,Corneal endothelium and anterior chamber depth did not meet ICL surgery needs(corneal endothelium ≥2000/mm^2^, anterior chamber depth ≥2.80mm),Severe refractive media clouding such as corneal leucoma, cataract and so on ,Serious complications during the perioperative period such as intraocular infection, lens damage, or intraocular hemorrhage ,Serious systemic diseases such as diabetes, systemic immune system diseases and so on. This study was approved by the Medical Ethics Committee of West China Fourth Hospital of Sichuan University (Registration number: HXSY-EC-2023040). All patients and their families agreed to participate and signed the written informed consent. The study was conducted in accordance with the Declaration of Helsinki. Surgical intervals are based on the participant's wishes. Participants can choose from 1-day or 1-week intervals between bilateral eye surgeries. This is the protocol used during the daily clinical practice. The data of patients who underwent bilateral ICL surgery were divided into two groups: 1 group had second eye surgery with a 1-day interval (74 people), and the other with a 1-week interval (46 people). The first eye surgery was conducted on more visually impaired eye.

### Surgical methods

Levofloxacin hydrochloride (0.5%, Santen) was used to dot both eyes 1 day before surgery. Pupils were dilated with compound tropicamide(1ml:5mg, Santen) 1 hour before surgery. Oxybuprocaine hydrochloride(20ml:80mg, Santen) eye drops were given 10 min before surgery. After flushing the conjunctival sac during the surgery, the operated eye was supplemented with 1–2 drops of oxybuprocaine hydrochloride. If an astigmatism ICL lens was to be implanted, corneal limbal marking was performed on the morning of surgery using a 4.5-gauge needle and a slit lamp.

ICL V4c lenses were implanted in the eyes of every participant. Standard disinfection was performed during the surgery, and the surgical eye was exposed. A 2.75 mm incision was made at the upper corneal limbus, and the ICL was loaded onto a specialized injector, injected, and unfolded through the corneal incision. A viscoelastic substance (1.7%, Bausch Lomb)was injected into the anterior chamber, and the ICL position was adjusted behind the iris using a positioning hook. Lens positioning markers had to align precisely with corneal limbus markers for toric lenses. The viscoelastic agent was then removed completely. After the pupil reduction by carbachol injection(1ml:0.1mg, Bausch Lomb), the incision was closed with an aqueous solution. The conjunctival sac was coated with tobramycin dexamethasone eye ointment(tobramycin 0.3%, dexamethasone 0.1%,Alcon), and the operated eye was covered with sterile dressings. All procedures were conducted by the same ICL specialist.

### Evaluation indicators

#### General information

Age, gender, education level score (primary school: 0 points; junior high school: 1 point; high school: 2 points; junior college: 3 points; undergraduate: 4 points; master: 5 points; doctor and above 6 points), childbearing for females (childlessness, cesarean birth, and normal delivery).

#### Preoperative anxiety level

The visual analog scale (VAS) anxiety scale was used to evaluate this before surgery. A 10 cm long horizontal line was drawn on paper; the left side of the line was zero (without any anxiety), and the right side was ten (extreme anxiety). The scale indicated increasing anxiety in order from left to right. In the surgical waiting room, the patient was instructed to point out the anxiety position on the horizontal line based on their inner feelings [12].

#### Pain

After surgery, pain indicators were collected using the VAS pain scale, similar to the VAS anxiety scale [13, 14].

#### Estimated operative time

The patient was asked to estimate the duration of the intraoperative period to assess the patient's self-perceived pain and anxiety.

#### Intraoperative cooperation and surgical time

The patient was extremely cooperative: 0 points; the patient was cooperative, but with occasional eye closure: 1 point; the patient was relatively cooperative, with eye rotation and eye closure: 2 points; and the patient was poorly cooperative, with head movement, eye closure, and eye rotation: 3 points. The intraoperative time taken (from the time the patient's eye was exposed to the ophthalmic microsurgical instruments to the time the patch was removed at the end of the procedure) was also recorded.

#### Patient satisfaction

It was measured by the VAS scale, following the same method as before.

#### MCP-1 level

About 100-200ul aqueous humor samples from the anterior chamber via the transparent corneal incision were obtained by 1ml injection syringe during first-eye and second eye surgery. Enzyme linked immunosorbent assay(ELISA) was used to measure MCP-1 concentration. All procedures were conducted under the instructions of the ELISA kit (Germany, Millipore). The specific steps were as follows: after incubating the sample with the captured antibody-coupled magnetic beads for 30 min, the samples were washed three times in the washing station. Then, the biotinylated detected antibody was added to each well and incubated in the dark for 1 h. Captured analytes were detected by adding streptavidin-phycoerythrin. Milliplex Analyte (5.1.0.0)was employed for the analysis.

### Statistical methods

SPSS V 19.0 (IBM) was used for statistical analyses. If the data conformed to a normal distribution, two independent samples t-test was applied for comparison between groups, and two paired samples t-test was applied for comparison between the two eyes within the group (age, time consumed in surgery, preoperative tension, satisfaction, estimated time of surgery, and MCP1). Otherwise, the rank-sum test was applied (intra-operative cooperation level, level of pain and recalled level of pain in the first eye, educational level). The chi-square test was used for gender. A non-parametric test with multiple independent samples was used for females to explore the relationship between fertility status and pain. The value of p less than 0.05 indicated a statistically significant difference.

## Results

### Baseline characteristics

This study included a total of 120 patients (39 males and 81 females). In the 1-day interval group, male/female is 22/52,average age is 25.24±5.00. In the 1-week interval group, male/female is 17/29,average age is 25.39±5.57.The comparison between the two groups showed no statistical differences in demographic data and the indicator of the first eye between the two groups. The baseline of the two groups was consistent. See Table 1.

**Table 1:** Baseline data of patients

**Baseline data**	**1-day interval group**	**1-week interval group**	***t*/*χ***^**2**^**/Z**	***P***
Cases	74	46		
Male/Female	22/52	17/29	0.675	0.411
Site of 1st surgery (Right/Left)	32/42	19/27	0.044	0.835
Age $(\overline{x } \pm s, {\text{y}})$	25.24±5.00	25.39±5.57	-0.151	0.880
Education level	4(4,4)	4(3.75,4)	-0.710	0.477
Anxiety of the 1st eye $(\overline{x } \pm s)$	3.42±1.99	3.65±1.82	-0.646	0.520
Pain of the 1st eye	2(1,2)	1(1,3)	-0.033	0.973
Satisfaction of the 1st eye $(\overline{x } \pm s)$	8.39±1.52	8.22±1.65	0.591	0.555
Estimated surgical duration of the 1st $(\overline{x } \pm s, {\text{min}})$	4.82±1.98	4.76±2.05	0.169	0.866
Cooperation level of the 1st eye	0(0,1)	0(0,1)	-0.154	0.878
Surgical duration of the 1st eye $(\overline{x } \pm s, {\text{min}})$	8.80±1.49	8.87±1.64	-0.248	0.804
MCP1 of the 1st eye (pg/ml)	861.27±114.06	896.91±106.64	-1.706	0.091

### Preoperative anxiety level

The anxiety scores of the first eye in the 1-day interval group and the 1-week interval group were 3.42±1.99 and 3.65±1.82, respectively, with no statistical difference between the two groups. The anxiety levels of the second eye decreased in both groups, reaching 2.80±1.81 and 2.89±1.68, respectively, showing statistical differences compared to the first eye. There was no statistical difference between second eye in the two groups. See Table 2.

**Table 2:** Comparison of characteristics between 1st and 2nd eye ICL surgery

**Parameter**	**1st eye**	**2nd eye**	**t/z**	***P***
Anxiety in the 1-day interval group $(\overline{x } \pm s)$	3.42±1.99	2.80±1.81	3.374	<0.001
Anxiety in the 1-week interval group $(\overline{x } \pm s)$	3.65±1.82	2.89±1.68	3.295	<0.001
t	-0.646	-0.285		
p	0.520	0.776		
Pain in the 1-day interval group	2(1,2)	2(1,4)	-4.416	<0.001
Pain in the 1-week interval group	1(1,3)	3(2,4.5)	-5.117	<0.001
z	-0.033	-1.978		
P	0.973	0.048		
Satisfaction in the 1-day interval group $(\overline{x } \pm s)$	8.39±1.52	7.55±1.84	4.898	<0.001
Satisfaction in the 1-week interval group $(\overline{x } \pm s)$	8.22±1.65	7.20±1.95	3.113	<0.001
t	0.591	1.015		
p	0.555	0.312		
Estimated duration in the 1-day interval group $(\overline{x } \pm s, {\text{min}})$	4.82±1.98	5.65±2.59	-3.724	<0.001
Estimated duration in the 1-week interval group $(\overline{x } \pm s, {\text{min}})$	4.76±2.05	6.76±2.44	-7.063	<0.001
t	0.169	-2.338		
P	0.866	0.021		
Cooperation in the 1-day interval group	0(0,1)	1(0,1)	-3.134	<0.001
Cooperation in the 1-week interval group	0(0,1)	1(0,2)	-3.753	<0.001
Z	-0.154	-2.542		
p	0.878	0.011		
Surgical duration in the 1-day interval group $(\overline{x } \pm s, {\text{min}})$	8.80±1.49	8.91±1.39	-0.728	0.469
Surgical duration in the 1-week interval group $(\overline{x } \pm s, {\text{min}})$	8.87±1.64	9.13±1.67	-1.632	0.110
t	-0.248	-0.799		
p	0.804	0.426		
MCP1 in the 1-day interval group (pg/ml)	861.27±114.06	898.38±118.43	-1.964	0.053
MCP1 in the 1-week interval group (pg/ml)	896.91±106.64	974.77±102.53	-3.285	<0.001
t	-1.706	-3.612		
p	0.091	<0.001		

### Pain level

The pain score of the first eye in the 1-day interval group was 2 (1,2), and in the 1-week interval group was 1 (1,3). The pain level of the second eye increased in both groups to 2 (1,4) and 3 (2,4.5) separately, which was statistically different from the first eye. The pain level of the second eye was statistically greater in the 1-week interval group than in the other group. See Table 2.

### Intraoperative cooperation and surgical time

The scores of cooperation in the first eye surgery were 0 (0,1) in both groups, rising to 1 (0,1) in the second eye surgery in the 1-day interval group and 1 (0.2) in the 1-week interval group. There was a statistically significant difference between the two eyes in both groups. The second eye cooperation in the 1-week interval group was significantly worse than that in the 1-day interval group, with a statistically significant difference. The surgical time of the first eye in the two groups was 8.80±1.49 and 8.87±1.64 respectively, and for the second eye, it was 8.91±1.39 and 9.13±1.67 respectively. There was no statistically significant difference between the two groups in terms of surgical time and between bilateral surgical time in both groups. See Table 2.

### Satisfaction

The satisfaction of the first eye surgery in the two groups was 8.39±1.52 and 8.22±1.65, respectively. The satisfaction of the second eye decreased to a score of 7.55±1.84 in the 1-day interval group and 7.20±1.95 in the 1-week interval group. There was no statistical difference between the two groups. The scores were statistically different between bilateral surgeries in both groups. See Table 2.

### Estimated surgical duration

The estimated surgical time consumed in the first eye surgery was 4.82 ± 1.98 and 4.76 ± 2.05 in two groups separately. There was no statistical difference between the two groups. The estimated duration of the second eye surgery was longer, with a statistically significant difference in both groups. In the 1-day interval group, it was 5.65±2.59; in the 1-week interval group, it was 6.76±2.44. The estimated duration of the second eye surgery in the 1-week interval group was longer than that in the 1-day interval group, and the difference was statistically significant. See Table 2.

### Recalled pain of the first eye surgery

When evaluating the pain during the second eye surgery, the examinee was asked to recall the pain level from the first surgery to ascertain if memory loss occurred. The recalled pain score of the first eye in the 1-day interval group was 1 (1,2), and in the 1-week interval group was 1 (0.2). Both were lower than the actual pain score of the first eye. There was a statistical difference between the recalled and actual pain in the 1-week interval group. The two groups also had a statistically significant difference in recalled pain. The 1-week interval group was more inclined to underestimate the level of pain in the first eye. See Table 3.

**Table 3:** Comparison of actual pain and recalled pain in the first eye surgery

**Group**	**Pain of the 1st eye**	**Recalled pain of the 1st eye**	**z**	***p***
1-day interval group	2(1,2)	1(1,2)	-1.020	0.308
1-week interval group	1(1,3)	1(0,2)	-3.196	<0.001
z	-0.033	-2.158		
p	0.973	0.031		

### MCP-1 levels

Preoperative MCP-1 levels in the first eye were not statistically different between the two groups. The MCP-1 level in the second eye was higher than that in the first eye in both groups, and the difference was statistically significant in the 1-week interval group. The MCP-1 level in the second eye in the 1-week interval group was higher than that in the 1-day interval group, with a statistically significant difference.

### Relationship between general information and pain

Depending on whether the women gave birth or not, they were divided into the childless group (53 people ), the cesarean section group (20 people) and the normal delivery group (8 people). The result of the Kruskal-Wallis test was H = 33.943, *p* < 0.001, which indicates a difference in pain level among the three groups of patients. After a two-by-two comparison, there was a difference in pain level between the childless group and the cesarean section group (test statistic=29.976,adjusted *p*<0.001), between the childless group and the normal delivery group(test statistic=33.664,adjusted *p*<0.001). However, there was no difference between the normal birth group and cesarean section groups(test statistic=3.688,adjusted *p*=1.000).

### Adverse reactions

The main adverse reactions were temporary corneal edema and transient high intraocular pressure. No serious adverse reactions such as intraocular infection, lens damage, or intraocular hemorrhage occurred.

## Discussion

With the improvement of ICL surgical equipment and techniques, patients who undergo the surgery are increasingly concerned about their experience and satisfaction. ICL surgery generally uses topical anesthesia, which is fast, efficient, inexpensive, and can reduce the risk of nerve damage and respiratory depression [15–18]. Although superficial anesthetics are becoming increasingly varied and analgesic, achieving a pain-free intraoperative experience remains difficult. Pain decreases satisfaction and makes them less cooperative during the surgery, which not only affects the operator's operation but also increases the risk of the surgery. Some studies have found that patients with binocular cataracts have worse cooperation in the second eye surgery [19].

In previous studies on cataract surgery, it has been found that the second eye surgery is more painful [20, 21]. However, other studies have also shown no significant difference in pain perception between the two eyes [22, 23]. The VAS method can quickly and effectively assess patients' satisfaction, pain, and anxiety levels [24]. It takes less than a minute to evaluate once. Numerous previous studies have evaluated patients' intraoperative pain on the first day after surgery and found no significant difference between the degree of pain in both eyes [25]. However, we assessed the intraoperative pain of patients shortly following surgery, which is more accurate.

The results of our study showed that during the second eye surgery, the level of anxiety reduced, the level of pain increased, satisfaction decreased, and the estimated surgery time was prolonged in both groups. In the second eye surgery, the level of patients' cooperation decreased, but it did not increase the duration of the surgery. When asked to recollect the discomfort of the first eye operation, most patients, especially those with a 1-week interval, continue to underestimate the pain.

Pain is a somatic perception or psychological state related to physiological injury and is the integration of physiological and psychological aspects. In the bilateral surgery performed by the same surgeon, the sensation of pain should be similar [21]. There are two possible reasons why ICL surgery on the second eye is more painful: physiological and psychological factors. Physiological factors: Inflammation may be a contributing factor to pain [26]. A study pointed out that in patients undergoing bilateral cataract surgery, certain cytokines, such as MCP1 in the second eye, were significantly elevated after surgery in the first eye [27], suggesting that surgery in one eye may cause an inflammatory response similar to sympathetic uveitis in the other eye. In cataract surgery, JiangL et al. found a positive correlation between pain and MCP-1 concentration in the aqueous humor [28]. Moreover, the analgesic drug may cause pharmacological tolerance. URsea et al. anticipated that the patient's tolerance to the anesthetic administered during the first eye surgery would render the anesthesia ineffective during the second eye surgery, increasing discomfort [6]. Psychological factors: previous studies have found that the level of intraoperative pain is related to the patient's mental state [29, 30]. In preparation for the first eye surgery, most patients are nervous and anxious, anticipating a high pain level. A certain degree of preoperative tension and anxiety can raise the patient's sensory threshold [31] and cooperation. During the surgery, patients doesn’t feel as painful as they expect so they feel that the first eye surgery is not particularly painful and have high satisfaction. When the second eye ICL surgery is performed, the patients are already familiar with the surgical procedure, the level of anxiety and tension has decreased [32], and the patients anticipate a relaxing and painless surgical experience. If the intraoperative experience was found to be inconsistent with their expectations, they experienced more pain and had less satisfaction [9, 31, 33]^.^ This is consistent with our findings that patients were less nervous, less cooperative and had higher pain levels and lower satisfaction, longer estimated surgical time during the second eye surgery. Furthermore, the patient's monocular vision improved immediately after the first eye surgery, resulting in higher expectations for the second eye. If they experience some pain during the operation, they believe the anesthesia was not as effective as in the first surgery, leading to more pain, less satisfaction, and poorer cooperation [34, 35]. Lastly, by the time a patient undergoes a second eye surgery, the agony of the first eye surgery has been forgotten. Patients tend to compare the more intense pain they just experienced with the lower pain in their memory, thus resulting in more pain [7]^.^This is consistent with our finding that a significant proportion of patients underestimated the pain in the first eye and that the longer the interval between two eyes was, the more obvious the forgetting was.

Currently, there are few studies analyzing whether the interval between bilateral surgeries affects the degree of pain. Our study found statistically significant differences between the two groups' second eye regarding estimated surgical duration, MCP1, cooperation, pain, and recalled pain of the first eye. The group with a 1-week interval had more pronounced pain, poorer cooperation, and longer estimated surgical time in the second eye surgery, suggesting that 1-week intervals should be avoided if possible. 1-week interval group also had a tendency to underestimate and forget the pain experienced during the first eye surgery following the procedure on their second eye.

Our study used ELISA to detect the concentration of MCP-1 in the aqueous humor of patients and analyzed the correlation between intervals and the expression level of MCP-1 in the contralateral eye. Previous studies have reported that the worsening pain in the second eye may be related to sympathetic uveitis caused by changes in inflammatory factors [36]. MCP-1 is the most important factor causing pain [37]. This factor exerts biological activity by binding it to its specific receptor, CCR2. Studies have found that blocking MCP-1 or its homologous receptor CCR2, as well as knocking out the CCR2 gene in mice, can alleviate pain after nerve injury [38]. Research has found that the level of MCP-1 in the aqueous humor significantly increases after surgery on the first eye [27, 38]. Our results show that the preoperative levels of MCP-1 in the second eye in both groups were higher than those in the first eye. This difference was statistically significant in the 1-week interval group. The MCP1 level in the second eye in the 1-week interval group was higher than that in the 1-day interval group, with a statistically significant difference. Previous study has reported that delayed immune responses may cause an increase in inflammatory factors after a certain period [39]. Our outcome is consistent with previous studies. Previous study has reported that the levels of TNF-α and IL-1β increase significantly after surgery on the first eye, which can serve as predictive factors for pain [40–42].

According to other clinical studies, there is a significant difference in pain tolerance before and after childbirth (especially vaginal delivery) for women. The difference in pain tolerance between younger women (who have not given birth) and older women (who have given birth naturally ) may not be due to age but may be related to childbirth history. Our study found that the childless group had the highest level of intraoperative pain, and the difference was statistically significant. The lowest pain level was found in the normal delivery group, and the middle level was found in the cesarean section group. However, there was no statistically significant difference between the two groups. This finding suggests that childbearing, especially normal delivery, may increase pain tolerance. However, additional research is required to determine if childbearing or age contributes to the effect and to determine the precise mechanism.

The shortcomings of our study: First, the sample size was small, with a higher proportion of female patients, which may have affected the accuracy of the study. Second, the interval between the two eye surgeries was divided into two groups without further grouping. Third, except for certain objective indicators, this study mainly focused on the subjective experience of patients, which introduces a certain level of subjectivity.

## Conclusions

In summary, the patient's anxiety decreased, the pain level increased, satisfaction decreased, cooperation worsened, and aqueous humor MCP1 increased following the second eye ICL surgery. The difference in the 1-week interval group was more significant than that in the 1-day interval group. Patients' intraoperative pain and tension can influence ophthalmic surgery more than other surgeries [43, 44] and are related to the success of surgery [21, 45, 46]. Currently, many studies are discussing how to control these psychological factors [47, 48]. Preoperative evaluation and education of patients can reduce intraoperative pain and minimize surgical risks [28]. It is recommended to provide psychological intervention before the second eye surgery, informing the patient in advance that the second eye is more painful and giving them a certain level of tension [49]. Additional intraoperative anesthesia can be considered in some patients. To improve patients' cooperation, satisfaction, and comfort, we recommend avoiding a 1-week interval when planning the surgical schedule.

## Data Availability

The datasets are available from the corresponding author on reasonable request.

## Abbreviations

ICL: Implantable Collamer Lens
VAS: Visual Analogue Scale
MCP-1: Monocyte chemoattractant protein-1
ELISA: Enzyme linked immunosorbent assay
M: Male
F: Female
R: Right eye
L: Left eye
